# The Impact of Fermentation on Aflatoxin and Fumonisin Levels in Sorghum Kernels and Flour Used to Prepare Complementary Food in Kerio Valley, Kenya

**DOI:** 10.1002/fsn3.4575

**Published:** 2024-11-15

**Authors:** Lmeriai Lesuuda, Meshack Amos Obonyo, Maureen Jepkorir Cheserek

**Affiliations:** ^1^ Department of Human Nutrition, Faculty of Health Sciences Egerton University Nakuru Kenya; ^2^ Department of Biochemistry and Molecular Biology, Faculty of Science Egerton University Nakuru Kenya

**Keywords:** aflatoxin, complementary foods, fermentation, fumonisin, sorghum

## Abstract

Sorghum is a major ingredient used in the production of complementary foods in Kenya's drylands, particularly in areas like Kerio Valley. However, it is known to be susceptible to aflatoxin and fumonisin contamination, which have adverse effects on human health. The current study aimed to assess the levels of aflatoxin and fumonisin in sorghum kernels and flour from Kerio Valley and to investigate whether fermentation (spontaneous or innoculum facilated) could reduce the levels of toxins. The sorghum samples were obtained through a cross‐sectional survey and subjected to ELISA analysis for quantification of aflatoxin and fumonisin levels. The aflatoxin levels ranged from 0 to 119.91 ppb for sorghum kernels and from 2.70 to 89.36 ppb in the flour, while fumonisin concentrations ranged from 0 to 30.65 ppm in sorghum kernels and 0.22 to 27.27 ppm in the flour. Fermentation significantly reduced the levels of the toxins in sorghum samples (*p* ≤ 0.05). The type of fermentation (*p* = 0.001), sample fermented (kernels or flour) (*p* = 0.004), and duration of fermentation (*p* = 0.037) significantly impacted the reduction of both toxins. Therefore, there is a need to integrate the adoption of fermentation with other effective postharvest practices to mitigate mycotoxin contamination in sorghum and other cereal‐based foods. In addition to the customary health and nutrition messages, promoting proper food handling, storage, and processing can significantly contribute to improving food safety and the overall health and nutritional status of young children.

## Graphical abstract

Sorghum kernels and flour used to prepare complementary foods in Kerio Valley were contaminated by high aflatoxin and fumonisin levels. High toxins levels are attributable to poor post‐harvest handling and storage practices of sorghum by the caregivers of young children. We explored whether, fermentation‐ an age old food processing practice, has a potential to render contaminated sorghum kernels and flour safe for human consumption.

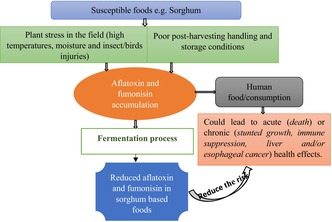

## 1 Introduction

Sorghum cultivation is prevalent in drought‐prone regions of Kenya and forms a significant portion of the diet for many households in these areas (Orr et al. 2016). Commonly processed into flour, sorghum is used either on its own or in combination with other flour to prepare complementary food (Mbijiwe, Ndung'u, and Kinyuru 2021). To promote its high nutritional value and ability to thrive in drylands, the Kenyan government and other development partners have been encouraging sorghum production among smallholder farmers in arid regions (Okeyo et al. 2020), such as the Kerio Valley, where erratic rainfall and persistent food and nutrition challenges are recurrent (Elgeyo Marakwet County 2018). As part of efforts to address these challenges, the Elgeyo Marakwet County government, in collaboration with a USAID‐funded project (Accelerated Value Chain Development—Drought Tolerant Crops), has promoted the production and consumption of sorghum and other drought‐tolerant crops in the area. This has led to sorghum becoming a fundamental ingredient in the preparation of complementary foods in the region (Kiome et al. 2021).

With increased utilization of sorghum and other drought‐tolerant food crops in the Kerio Valley, it was anticipated that child nutrition outcomes would improve. However, Kipyego and Mugalavai (2019) reported high rates of childhood malnutrition, particularly among households targeted with drought‐tolerant crop interventions. Their study indicated a stunting rate of 67%, significantly exceeding both the county's (22%) and national (18%) stunting levels (KNBS and ICF 2023). Based on this finding, it is hypothesized that, among other causal factors, the consumption of mycotoxin‐contaminated foods could be a major contributor to the high prevalence of childhood malnutrition rates in the Kerio Valley.

Aflatoxin and fumonisin are detrimental fungal by‐ products that contaminate food crops in the field and during storage conditions that favour proliferation of fungi (Obonyo and Salano 2018). Globally, approximately 60%–80% of staple foods for humans, including cereal‐based complementary foods, are contaminated by mycotoxins (Eskola et al. 2020; Ojuri et al. 2019). This contamination can have significant effects on young children due to their lower body mass, higher metabolic rate, underdeveloped organ functions, and limited detoxification mechanisms (Kimanya et al. 2014). As a result, exposure to both aflatoxin and fumonisin can significantly contribute to childhood stunting (Mupunga, Mngqawa, and Katerere 2017). This is attributed to the mycotoxins' capacity to reduce the absorptive surface area and barrier function of the small intestine (Knipstein et al. 2015), as well as weaken the immune system (Leroy, Wang, and Jones 2015). Elevated levels of aflatoxin and fumonisin in sorghum have been documented in various regions of Kenya (Kang'ethe et al. 2017; Sirma et al. 2021). As a result, the use of sorghum in complementary foods (Kiome et al. 2021; Lesuuda, Obonyo, and Cheserek 2021) could potentially expose young children to different mycotoxins, possibly contributing to poor child growth in Kerio Valley.

A lack of awareness about mycotoxins among farmers, as well as poor agronomic practices and hot and dry climates, can lead to increased fungal growth and mycotoxin production in certain food commodities (Makun 2013). Our recent study (Lesuuda, Obonyo, and Cheserek 2021) revealed that caregivers growing sorghum in Kerio Valley had limited knowledge about mycotoxin contamination, and their postharvest handling and storage practices for sorghum were subpar. This underscores the importance of assessing aflatoxin and fumonisin levels in sorghum kernels and flour to better inform efforts to reduce dietary mycotoxin exposure and enhance the safety of complementary foods.

## 2 Methods

### 2.1 Ethical Approval

The study was carried out according to the revised (2000) Helsinki Study Protocols of 1975 and approved by the Egerton University Research Ethics Committee (EUREC/APP/130/2021). Permission to carry out the study was granted by the Kenya National Commission for Science, Technology, and Innovation (License No: NACOSTI/P/21/11631) and the Elgeyo Marakwet County leadership. Informed consent was sought from targeted farmers before sample collection.

### 2.2 Study Area and Sample Collection Procedure

In June and July 2020, sorghum samples were collected from Kerio Valley's three main sorghum‐growing wards (Endo, Emsoo, and Arror). The sample collection process involved random selection of 30 villages. Targeting households with children aged 6–23 months of age, 120 sorghum‐growing households were randomly selected, and samples were collected from each household. This structured sampling approach ensured a representative collection of sorghum samples.

Following procedures used by Obade et al. (2015), 1 kg of sorghum kernels and flour were collected from each of the selected households. Through this, kernels and flour samples were scooped at different points from storage bags and combined to get a representative sample. Samples from each collection point (household) were kept separately and stored in a cool box using labeled brown khaki bags and conveyed for mycotoxin analyses at Egerton University.

### 2.3 Isolation and Identification of Fungi in Sorghum Kernel and Flour

In order to estimate the impact of produce handling practices, we analsysed the fungal community in the collected sorghum samples. As per the protocol followed by Owuor et al. (2017), for fungal isolation, sorghum kernels were first surface sterilized in 1.3% sodium hypochlorite and then rinsed three times for two minutes with sterile distilled water. Fifteen kernels were placed on Petri dishes (90 × 15 mm) containing Potato Dextrose Agar (PDA, Hi Media) supplemented with 50 mg of streptomycin sulphate per liter of medium. To ensure consistency in the experimental outcomes, the procedure was replicated three times for each sample.

The dilution plate technique was employed for analyzing flour samples. One gram of flour was combined with 9 mL of sterile distilled water and diluted to a concentration of 1 × 10^−3^. Subsequently, 1 mL of the diluted solution was uniformly spread onto duplicate plates containing PDA (amended with 50 mg of streptomycin sulphate per liter of medium). Following this, the plates with samples were placed in an incubator for 5–7 days at 25°C, subjected to 12 h of daylight and darkness cycles. After the incubation period, the total number of plates with infected samples and the quantity of fungal colonies per plate were recorded.

The fungal colonies resulting from the inoculation were individually subcultured to obtain pure cultures for microscopic identification and cultural characterization. For the microscopic identification of *Fusarium* isolates, their colonies were cultured on a synthetic nutrient agar (SNA) medium at room temperature for 7–14 days to enable sporulation. Moreover, *Fusarium* colonies subcultured on PDA were used for cultural classification. Manuals developed by Nelson, Toussoun, and Marasas (1983) and Jenkinson and Parry (1994) were utilized to identify distinct species of *Fusarium*. Other fungal genera, were subcultured on amended PDA and identified based on specific species using the cultural and morphological features outlined by Klich (2002). Finally, the frequency of fungal genera and the relative percentage of specific species within a genus of fungi were calculated as per the protocols described by Ghiasian et al. (2004).

The frequency is calculated as the number of samples infected with fungi divided by the total number of samples analyzed, multiplied by 100.

Relative percentage is calculated as the number of fungal species isolated divided by the total number of fungal isolated, multiplied by 100.

### 2.4 Analysis of Mycotoxin Levels in Sorghum Samples

The Enzyme‐Linked ImmunoSorbent (ELISA) Assay technique was used to quamtify aflatoxin and fumonisin levels in sorghum samples. Sorghum kernels were ground to get 200 g of fine flour. A homogenous sample was obtained by carefully mixing each sample. Then aflatoxins quantification was carried out using the Eurofins *Celer AFLA* ELISA kit as per the manufacturer's procedures. In addition, fumonisin toxins were extracted from 20 g of well‐mixed samples, and their levels were quantified according to the ELISA kit manufacturer's protocol (Helica Biosystems Inc., Fullerton, CA). The ELISA microplate reader's (ThermoScientific) output was read at 450 nm, and its values and kits' standards were used to generate the standard curves. The toxin detection kit's standard limits for aflatoxin and fumonisin were 0–80 ppb and 0–6 ppm, respectively. Samples that had toxin levels exceeding these standards were serially diluted following the manufacturer's instructions, and the resulting values were adjusted by the dilution factor.

### 2.5 Fermentation of Sorghum Kernels and Flour

Randomly selected sorghum kernels and flour samples were divided into two proportions of 200 g each. The portions were fermented according to Mukandungutse et al. (2019) protocols, where the samples were mixed with sterile distilled water in a ratio of 1:1.5 (w/v). One portion of each sample (kernel and flour) was allowed to spontaneously ferment under room temperature for 24, 48, and 72 h, respectively, where samples were withdrawn and dried. The second portion was fermented with the addition of *Lactobacillus plantarum*. The bacterial strain was resuscitated according to the protocol used by Ntsamo et al. (2020). Sorghum slurries were inoculated with 3 mL of suspension of *Lb. plantarum* to achieve a final concentration of 10^−7^ CFU/g, well‐mixed, and incubated at 37°C for 24, 48, and 72 h. After each fermentation duration, according to Kurniadi et al. (2019), samples were put in the dryer cabinet at 60°C for 16 h for adequate drying. Dried samples were then grounded into a fine powder and analyzed for aflatoxin and fumonisin using the ELISA method.

### 2.6 Data Analysis

The analysis involved assessing the number of fungal‐infected samples and the relative density (%) of isolates per species. Statistical Package for Social Software (SPSS) version 26 was utilized for the analyses. The comparison of mean values for aflatoxin and fumonisin in sorghum samples employed the student *t*‐test, while analysis of variance (ANOVA) was used to calculate aflatoxin and fumonisins mean in sorghum samples from three different wards. Additionally, fermentation data underwent a two‐way analysis of variance using the general linear model (GLM) procedures. Tukey's test was performed to distinguish means, and a significance level of *p* < 0.05 was applied. Qualitative data from the observation guide was used to explain the levels of aflatoxin and fumonisin found in sorghum samples.

## 3 Result

### 3.1 Characterization of Fungal Isolates From Sorghum Samples

*Fusarium* and *Aspergillu*s were the predominant species, although their occurrence and abundance varied between sorghum kernel and flour (Table 1). *Fusarium* genera were the most common in sorghum kernels, with a frequency of 90.8% and a relative percentage of 43.3%, followed by *Aspergillus* genera. Conversely, *Aspergillus* genera were more dominant in sorghum flour, with a frequency of 87.5% and a relative percentage of 54.4%. *Fusarium* was the second most dominant fungal genera in sorghum flour, with a frequency of 60.8% and a relative percentage of 23.1%. Although other fungal genera were present in the sorghum samples, *Fusarium* and *Aspergillu*s were further analyzed at the species level due to their association with aflatoxin and fumonisin production. This analysis revealed that *Aspergillus flavus* (28.3%) and *Parasiticus* (23.3%) dominated sorghum kernels, while *A. fumatus* (56.7%) and *flavus* (41.7%) were most common in sorghum flour. Additionally, within the *Fusarium* family, *p* *roliferatum* and *v* *erticilloide* were the most predominant species in both sorghum kernels and flour (Tables 2 and 3).

**TABLE 1:** Fungal species isolated from sorghum kernel and flour.

No	Fungus genera	Sorghum kernel			Sorghum flour		
		Number isolated	Relative (%)	Frequency (%)	Number isolated	Relative (%)	Frequency (%)
1.	*Aspergillus*	203	17.55	51.67	691	54.4	87.50
2.	*Fusarium*	501	43.30	90.83	115	9.05	17.50
3.	*Penicilium*	105	9.08	36.67	23	1.81	14.17
4.	*Mucor*	37	3.20	25.83	294	23.13	60.00
5.	*Curvularia*	40	3.46	19.17	24	1.89	10.83
6.	*Alternaria*	60	5.19	31.67	16	1.26	21.67
7.	*Chaetomium*	30	2.59	15.83	31	2.44	17.50
8.	*Phoma*	33	2.85	13.33	10	0.79	26.67
9.	*Cladosporium*	46	3.98	15.00	18	1.42	37.50
10.	*Nigrospora*	0	0.00	0.00	21	1.65	15.00
11.	*Epicoccum*	8	0.69	1.67	0	0.00	0.00
12.	*Verticillium*	17	1.47	10.83	13	1.02	16.67
13.	*Trichoderma*	1	0.09	0.83	6	0.47	5.83
14.	*Fusidium*	76	6.57	3.33	9	0.71	4.17

**TABLE 2:** The frequency and relative percentage of fungal species isolated from sorghum kernels.

No	Name of the fungus	Total number of isolates	Frequency (%)	Relative percentage
1.	*Aspergillus niger*	39	14.2	4.61
2.	*Aspergillus flavus*	74	28.3	8.74
3.	*Aspergillus parasiticus*	82	23.3	9.69
4.	*Aspergillus fumigatus*	8	4.2	0.95
5.	*Fusarium proliferatum*	292	69.2	34.52
6.	*Fusarium verticilloides*	91	34.2	10.76
7.	*Fusarium solani*	51	33.3	6.03
8.	*Fusarium oxysporum*	58	20.0	6.86
9.	*Fusarium keratiti*	9	0.8	0.01

**TABLE 3:** The frequency and relative percentage of fungal species isolated from sorghum flour samples.

No	Name of the fungus	Number of isolates	Frequency (%)	Relative percentage
1.	*Aspergillus parasiticus*	106	32.5	10.2
2.	*Aspergillus flavus*	153	41.7	14.7
3.	*Aspergillus niger*	55	15	5.3
4.	*Aspergillus fumigatus*	281	56.7	27.0
5.	*Aspergillus lentulu*	6	4.2	0.6
6.	*Aspergillus tereus*	46	15.8	4.4
7.	*Aspergillus tamari*	19	8.3	1.8
8.	*Aspergillus candidus*	25	5.8	2.4
9.	*Fusarium proliferatum*	144	38.3	13.8
10.	*Fusarium verticilloides*	47	24.2	2.6
11.	*Fusarium solani*	68	11.7	6.5
12.	*Fusarium oxysporum*	3	16.7	5.0
13.	*Fusarium keratitis*	52	2.5	0.3

### 3.2 Aflatoxin in Sorghum Kernel and Flour

In the 60 samples analyzed, 98.3% of the sorghum kernels and 100% of the flour contained detectable levels of aflatoxin. Moreover, 83.3% of the kernels and 96.7% of flour samples had aflatoxin levels above the 10 ppb limit. Sorghum kernel had a wide range of aflatoxin levels (0–119.91 ppb) compared with those observed in flour samples (2.70–89.36 ppb). Endo ward recorded the highest levels of aflatoxin in kernels with a mean value of 42.21 ± 21.26 ppb, with Arror ward having the lowest mean value (17.42 ± 12.11 ppb). Further, Emsoo ward had the highest mean level for total aflatoxin in flour (43.45 ± 20.00 ppb) compared with other wards (Table 4). Overall, flour samples had the highest aflatoxin mean value (36.72 ± 19.50 ppb).

**TABLE 4:** Aflatoxin in sorghum kernel and flour from the three survey areas.

Ward	Sorghum samples (*n*)	Positive samples (%)	Range (ppb)	Mean ± SD (ppb)#	*F*	*p*
Arror	Kernel (20)	31.7	0–38.65	17.42 ± 12.11^a^	7.28	0.002*
Endo	Kernel (20)	33.3	5.87–84.35	42.21 ± 21.26^ab^		
Emsoo	Kernel (20)	33.3	3.83–119.91	41.95 ± 32.77^ab^		
Total	60	59 (98.3)	0–119.91	33.85 ± 26.00		
Arror	Flour (20)	33.3	2.70–55.28	25.17 ± 10.24^a^	6.25	0.004*
Endo	Flour (20)	33.3	17.87–89.36	41.54 ± 21.55^b^		
Emsoo	Flour (20)	33.3	8.46–87.43	43.45 ± 20.00^b^		
Total	60	100	2.70–89.36	36.72 ± 19.50		

^#^ Data are mean ± standard deviation, *p* < 0.05, significant by analysis of variance (ANOVA).

* Means with the same letters (by column) are not significantly different.

### 3.3 Fumonisin in Sorghum Kernel and Flour

All sorghum flour (100%) and 98.3% of the kernel samples had fumonisins above the regulatory limit of 1 ppm. Although the difference was not significant, sorghum kernels had the highest mean value (12.90 ± 8.07) for fumonisins over the flour samples (10.04 ± 7.21). Additionally, compared with other wards, Endo ward recorded the highest mean value for fumonisin levels in both sorghum kernel (14.00 ± 6.74 ppm) and flour (11.68 ± 7.62 ppm) (Table 5).

**TABLE 5:** Fumonisin levels in sorghum kernel and flour.

Ward	Sorghum samples (*n*)	Positive samples (%)	Range (ppm)	Mean ± SD (ppm)^a^	*F*	*p*
Arror	Kernel (20)	30.1	0–25.06	11.84 ± 8.25	0.34	0.707
Endo	Kernel (20)	33.3	7.88–25.48	14.00 ± 6.74		
Emsoo	Kernel (20)	33.3	3.02–30.65	12.87 ± 9.29		
Total	60	58 (96.7)	0–30.65	12.90 ± 8.07		
Arror	Flour (20)	33.3	2.13–20.39	10.90 ± 6.91	0.125	0.883
Endo	Flour (20)	33.3	2.41–27.27	11.68 ± 7.62		
Emsoo	Flour (20)	33.3	0.22–25.02	10.55 ± 7.42		
Total	60	100	0.22–27.27	10.04 ± 7.21		

^a^ Data are mean ± standard deviation, *p* < 0.05, significant by analysis of variance (ANOVA).

### 3.4 Effects of Fermentation on Aflatoxin and Fumonisin Levels

**FIGURE 1:**
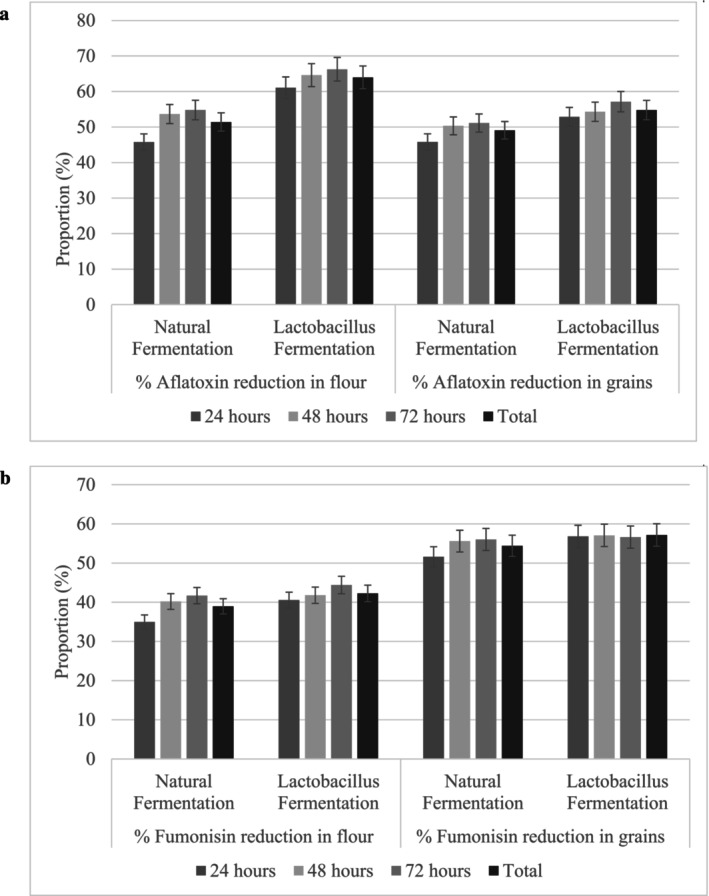
Reduction of aflatoxin and fumonisin levels in sorghum kernels and flour. (a) The percentage reduction in aflatoxin was significantly influenced by the type of fermentation (*F* = 24.287, *p* = 0.001), the type of sample (flour vs. kernels) (*F* = 9.706, *p* = 0.004), and fermentation duration (*F* = 3.690, *p* = 0.037). (b) Similarly, the percentage reduction of total fumonisin was significantly affected by the type of fermentation (*F* = 14.472, *p* = 0.001), the type of sample (flour vs. kernels) (*F* = 364.029, *p* < 0.001), and the fermentation duration (*F* = 8.542, *p* = 0.001).

The fermentation process significantly reduced aflatoxin and fumonisin levels in test samples. This led to a 54.82% and 48.19% average reduction for total aflatoxin and fumonisins respectively, after 72 h of fermentation. The addition of *Lactobacillus plantarum* bacteria during fermentation recorded better aflatoxin and fumonisin effects compared with fermenting sorghum samples without adding any bacteria (Figure 1). Overall aflatoxin was better reduced in flour samples compared with sorghum kernel, while fumonisin level was greatly reduced in sorghum kernel. An increase in fermentation time was associated with an accelerated reduction in toxin levels (*F* = 8.542, *p* = 0.001), Figure 1.

## 4 Discussion

This study revealed that sorghum kernel and flour samples collected from Kerio Valley, intended for household consumption, including the preparation of complementary foods, were highly contaminated by aflatoxin and fumonisin. This is relatable to the diverse genera of fungi isolated from sorghum kernel and flour (Tables 2 and 3). Similar to findings reported by Machio (2016) and Ackerman, Wenndt, and Boyles (2021), *Aspergillus* and *Fusarium*, which are known to produce aflatoxin and fumonisins, respectively, were the most dominant species among the fungal isolates.

Among the sorghum samples, flour had the highest mean value for total aflatoxin attributable to their post‐harvest handling and storage practices. The study respondents reported milling sorghum kernels in local milling facilities or traditional tools, which could have residual flour from the subsequent batches, which may be a source for cross‐contamination for flours (Ntuli et al. 2013). During focus group discussions, caregivers reported using pestle and mortars, which are not washed at any time, to mill sorghum kernel into flour (Lesuuda, Obonyo, and Cheserek 2021). The same observation was made by Beyene et al. (2016) in Ethiopia, where milling facilities were not frequently cleaned. The lack of proper sanitization of milling facilities could be one of the explanations for the high levels of aflatoxin in sorghum flour. To worsen the situation, the majority of caregivers are storing sorghum flour in plastic buckets, which are likely to promote fungal growth and mycotoxin contamination through heat and moisture retention (Mutegi et al. 2013). Comparable to the current study finding, other studies (Kihara 2015; Sirma et al. 2016) have reported high aflatoxin levels above regulatory limits in sorghum samples from other parts of the country.

The majority of sorghum samples in the present study exhibited detectable levels of fumonisin, consistent with the findings of Kang'ethe et al. (2017) (Table 5). Interestingly, sorghum kernels displayed higher mean fumonisin levels compared to flour samples. It is worth noting that the practice of sorting the kernels before milling, which was commonly reported by caregivers in the study area (Lesuuda, Obonyo, and Cheserek 2021), appeared to be associated with lower fumonisin levels in the flour. This practice was found to be more effective in reducing fumonisin levels compared to aflatoxin (Mutiga et al. 2014). In addition, Van der Westhuizen et al. (2011) suggested that visual sorting may not be sufficient to reduce aflatoxin levels as effectively as it does for fumonisin, emphasizing the importance of identifying critical control points for managing aflatoxin and fumonisin contamination in cereal‐based foods. It is noteworthy that the mean values for total aflatoxin and fumonisin in both sorghum samples in the present study exceeded the Kenya limits of 10 ppb for aflatoxin and the European Union legislation regulatatory limit of 1 ppm for fumonisin.

Mycotoxins threaten public health, food and nutrition security, trade, and the economy (N'dede et al. 2012). The costs of contamination are significant, encompassing health ramifications that lead to loss of labor and increased healthcare expenses, as extended exposure to aflatoxin, fumonisin, or both have been linked to heightened carcinogenicity (WHO 2018). Fumonisin exposure through the diet has been linked to esophageal cancer, a leading cause of cancer‐related deaths globally (Parker et al. 2010). In Western Kenya, where esophageal cancer is a major concern for both men and women (Odera et al. 2017), the presence of fumonisin in numerous food samples has been reported (Mutiga et al. 2015; Njeru et al. 2019), indicating chronic exposure to the toxin through food. Therefore, these findings suggest that dietary exposure to fumonisin may be contributing to the high incidence of esophageal cancer in Western Kenya (Patel et al. 2013). Furthermore, mycotoxins have been reported to cause health effects in animals, including livestock and poultry, as mycotoxins are potent hepatotoxins, immunosuppressants, mutagens, and carcinogens (Tola and Kebede 2016). As such, consumption of mycotoxin‐contaminated feed for extended periods could lead to reduced milk, beef, or wool quality in ruminants, resulting in financial losses for farmers and affecting their ability to afford food, healthcare, or education (Tola and Kebede 2016).

In Kenya, sorghum serves various purposes, including making leavened bread, fermented and non‐fermented porridge, and cakes (Ari et al. 2022). Notably, the beer industry has begun playing a significant role in its value chain for beer production (Ochieng 2011). This has sparked renewed interest in the commercial production of sorghum, offering farmers the potential for higher returns and access to new market channels. Additionally, heightened health concerns and awareness have contributed to a gradual increase in the use of sorghum products due to their nutritional value, as evidenced by the growing quantity and variety of processed sorghum items available at local supermarkets (Kilambya and Witwer 2013). Increased sorghum demand is expected to play a pivotal role in future industries (Ochieng 2011). However, the levels of aflatoxin and fumonisin reported in the present study and other parts of the country (Kang'ethe et al. 2017; Njeru et al. 2019) may hinder its potential uses and affect both households and the country's economic development. Food crops contaminated by mycotoxins are likely to face restrictions in international markets, leading to reduced income and purchasing power for farmers (Njeru et al. 2019). To evade this loss, farmers or traders may divert the rejected food crops and use them as animal feed (Mutegi, Cotty, and Bandyopadhyay 2018).

In Kenya, food safety standards allow the sale of feeds that test up to 100 ppb but recommend an ideal of 20 ppb (Sirma et al. 2018). Despite the existence of food safety standards, some traders disregard the regulatory limits and fetch a higher price for rejected foodstuffs in the informal food market than in the feed market, exposing relatively poor consumers to higher risks. In the event of a mycotoxin outbreak, both farmers and traders face significant challenges. For instance, during an aflatoxin alert in 2009, maize prices dropped by half, from 1800 Kenyan shillings to 900 Kenyan shillings in Kitui County (Marechera and Ndwiga 2015). Additionally, at least 2.3 million bags of maize were deemed unfit for human and livestock consumption or trade during the aflatoxin outbreak between 2004 and 2006 (Marechera and Ndwiga 2015). Despite multiple studies reporting aflatoxin and fumonisin prevalence in cereal‐based food crops, significant attention has been focused on regions with fatal mycotoxicosis outbreaks (Mutiga et al. 2015).

The study documented high toxin levels in sorghum samples, with caregivers' limited mycotoxin knowledge and subpar storage practices being identified as contributing factors (Lesuuda, Obonyo, and Cheserek 2021). Poor mycotoxin knowledge is associated with inadequate post‐harvest practices that promote fungal growth and toxin production in susceptible foods like sorghum (Makun 2013). Despite a large proportion of samples containing high toxin levels, samples from the Arror ward exhibited the lowest mean value compared to samples from other wards. This lower mean value in Arror may be attributed to better mycotoxin knowledge and storage practices among farmers (Lesuuda, Obonyo, and Cheserek 2021). Suboptimal postharvest handling, such as insufficient drying, and storage of high‐moisture grains in poorly aerated storage facilities heighten the risk of spoilage and mycotoxin contamination (Temba, Njobeh, and Kayitesi 2017). In Kerio Valley, farmers rely on traditional methods, including visual and sound tests, to assess kernel dryness, which are less accurate compared to moisture meters (Lesuuda, Obonyo, and Cheserek 2021). The use of these traditional methods may result in packaging and storing kernels with high moisture levels, leading to fungal growth and contamination (Hell et al. 2008).

The elevated levels of toxins in sorghum samples from Kerio Valley could be attributed to certain practices, such as drying and shelling the kernels on bare ground. When kernels are dried on bare ground, they come into contact with the soil, leading to an increase in moisture content through water absorption from the surface (Kamala et al. 2016). Storing inadequately dried kernels in traditional poorly ventilated granaries with leaking roofs, commonly found in Kerio Valley, could further elevate the heat and moisture content of the stored kernels, making them more susceptible to contamination. Moreover, placing storage bags directly on the floor may also contribute to increased water absorption. These practices, combined with the hot and dry climatic conditions of Kerio Valley, contribute to heightened mycotoxin contamination in foods (Taye et al. 2018). It's worth noting that households with lower monthly incomes have limited capacity to implement post‐harvest interventions that could mitigate mycotoxin contamination. Poverty has been linked to increased dietary exposure to mycotoxins (Leroy, Wang, and Jones 2015). Caregivers in the study area expressed reluctance to allocate their limited resources to post‐harvest techniques such as drying materials and hermetic bags, which could help reduce the risk of fungal exposure and mycotoxin contamination in their food crops. Additionally, poverty is correlated with poor‐quality diets (Hoffmann, Jones, and Leroy 2018), and some households from Emsoo ward mentioned consuming moldy sorghum kernels due to food shortages (Lesuuda, Obonyo, and Cheserek 2021). This underscores the heightened health risk from mycotoxin exposure faced by underprivileged households. Therefore, there is a pressing need for affordable and practical approaches, such as fermentation, to address this issue.

The process of fermentation is renowned for its ability to preserve and enhance the safety of food products. During fermentation, lactic acid is generated, leading to a decrease in the pH of the food. This acidic environment prolongs the shelf life of the fermented product by preventing the growth of harmful microorganisms (Tsafrakidou, Michaelidou, and Biliaderis 2020). Additionally, research has indicated that fermentation can notably reduce the presence of aflatoxin and fumonisin in maize (Mukandungutse et al. 2019). Despite these advantages, a limited number of caregivers in the three surveyed areas reported utilizing fermented sorghum products (Lesuuda, Obonyo, and Cheserek 2021). The study revealed that fermentation could effectively reduce aflatoxin and fumonisin levels in sorghum kernel and flour. However, it was observed that fermentation was more successful in reducing aflatoxin levels compared to fumonisin levels. This difference in efficacy is likely attributed to the distinct molecular structures of aflatoxin and fumonisin, which may impact their interaction with the by‐products produced during fermentation.

**FIGURE 2:**
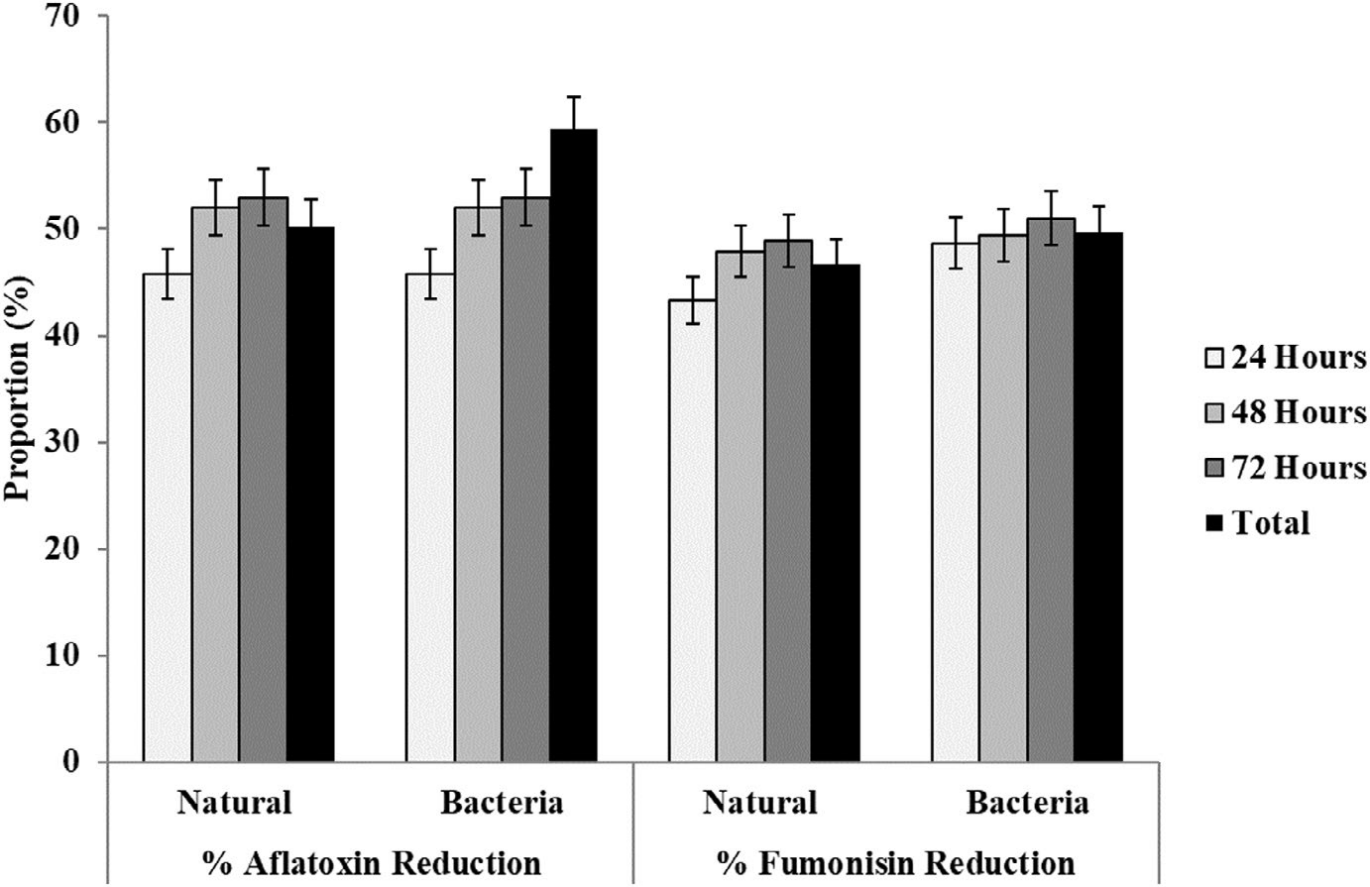
Percentage reduction of total aflatoxin and fumonisin over fermentation time, with and without *Lactobacillus plantarum*.

The study conducted a comparison of toxin reduction after fermentation between sorghum kernel and flour samples, revealing significant differences in the change of aflatoxin and fumonisin levels (Figure 2). Specifically, sorghum kernel showed a lower reduction in aflatoxin levels but a greater decrease in fumonisin levels compared to the flour samples. These differences can be attributed to the initial total toxin levels and microbial composition of the samples, both of which play a crucial role in determining the extent of mycotoxin reduction during fermentation. The initial microorganism composition in the samples significantly influences the production of microbial metabolites and their interactions with toxins (Adebo, Kayitesi, and Njobeh 2019).

The study observed that prolonged fermentation resulted in decreased levels of both aflatoxin and fumonisin, in line with findings from other research (Mukandungutse et al. 2019; Nyamete, Bennink, and Mugula 2016). This trend is likely attributable to increased production of lactic acid and other metabolites associated with mycotoxin reduction during extended fermentation periods (Nyamete, Bennink, and Mugula 2016; Adebo, Kayitesi, and Njobeh 2019). Notably, the use of *Lactobacillus plantarum* led to the highest mycotoxin reduction, possibly due to accelerated fermentation conditions compared to natural fermentation. However, natural fermentation also proved effective, achieving close to 50% reduction for both toxins (Figure 2). Other studies have similarly reported more than half a reduction in aflatoxin levels in maize through natural fermentation (Mukandungutse et al. 2019; Nyamete, Bennink, and Mugula 2016). The natural microorganisms in sorghum samples and high concentrations of bioactive compounds such as polyphenols, flavonoids, and tannins (Adebo, Kayitesi, and Njobeh 2019) might have contributed to the mycotoxin reduction in natural fermentation. In summary, fermentation is recognized as one of the simple and effective methods for reducing mycotoxins through endogenous enzymes and compounds produced by fermenting organisms (Okeke et al. 2018). Given its simplicity and affordability, fermentation remains a viable method for farmers to transform toxin‐contaminated cereal‐based foods into safe consumables.

## 5 Conclusion

The research emphasizes the significant presence of aflatoxin and fumonisin in sorghum kernels and flour, commonly used as ingredients in complementary foods in Kerio Valley. The detection of these toxins in food destined for infants and young children is particularly alarming, given their heightened susceptibility to adverse health effects. Traditional postharvest methods designed to prevent contamination are not always entirely successful in eliminating aflatoxins and fumonisins. The study has shown that fermentation can effectively reduce the levels of these toxins in sorghum by breaking down or transforming mycotoxins into less harmful compounds. By implementing these measures to address mycotoxin contamination in sorghum, we can greatly enhance the safety and nutritional value of complementary foods for young children.

## Author Contributions

**Lmeriai Lesuuda:** conceptualization (equal), data curation (equal), formal analysis (lead), funding acquisition (supporting), investigation (equal), methodology (equal), project administration (equal), resources (supporting), software (equal), supervision (supporting), validation (equal), visualization (equal), writing – original draft (lead), writing – review and editing (lead). **Meshack Amos Obonyo:** conceptualization (lead), data curation (lead), formal analysis (lead), funding acquisition (equal), investigation (equal), methodology (equal), project administration (lead), resources (lead), software (equal), supervision (equal), validation (equal), visualization (equal), writing – original draft (equal), writing – review and editing (equal). **Maureen Jepkorir Cheserek:** conceptualization (equal), data curation (equal), formal analysis (equal), funding acquisition (equal), investigation (lead), methodology (lead), project administration (equal), resources (equal), software (equal), supervision (lead), validation (lead), visualization (equal), writing – original draft (equal), writing – review and editing (equal).

## Conflicts of Interest

The authors declare no conflicts of interest.

## Data Availability

Due to ethical restrictions, datasets used and/or analyzed in this study are available upon a reasonable request.
